# Acute Ethanol Inhibition of γ Oscillations Is Mediated by Akt and GSK3β

**DOI:** 10.3389/fncel.2016.00189

**Published:** 2016-08-17

**Authors:** JianGang Wang, JingXi Zhao, ZhiHua Liu, FangLi Guo, Yali Wang, Xiaofang Wang, RuiLing Zhang, Martin Vreugdenhil, Chengbiao Lu

**Affiliations:** ^1^Key Laboratory for the Brain Research of Henan Province, Xinxiang Medical UniversityXinxiang, China; ^2^Department of Pathophysiology, Xinxiang Medical UniversityXinxiang, China; ^3^Psychiatric Hospital of Henan ProvinceXinxiang, China; ^4^Department of Neurobiology and Physiology, Xinxiang Medical UniversityXinxiang, China; ^5^Department of Psychology, Xinxiang Medical UniversityHenan, China; ^6^Department of Health Sciences, Birmingham City UniversityBirmingham, UK

**Keywords:** γ oscillation, dopamine receptor, Akt, GSk3β, PKA, NMDA receptor

## Abstract

Hippocampal network oscillations at gamma band frequency (γ, 30–80 Hz) are closely associated with higher brain functions such as learning and memory. Acute ethanol exposure at intoxicating concentrations (≥50 mM) impairs cognitive function. This study aimed to determine the effects and the mechanisms of acute ethanol exposure on γ oscillations in an *in vitro* model. Ethanol (25–100 mM) suppressed kainate-induced γ oscillations in CA3 area of the rat hippocampal slices, in a concentration-dependent, reversible manner. The ethanol-induced suppression was reduced by the D1R antagonist SCH23390 or the PKA inhibitor H89, was prevented by the Akt inhibitor triciribine or the GSk3β inhibitor SB415286, was enhanced by the NMDA receptor antagonist D-AP5, but was not affected by the MAPK inhibitor U0126 or PI3K inhibitor wortmanin. Our results indicate that the intracellular kinases Akt and GSk3β play a critical role in the ethanol-induced suppression of γ oscillations and reveal new cellular pathways involved in the ethanol-induced cognitive impairment.

## Introduction

Gamma (γ) oscillations arise from rhythmic synchronized synaptic activity in the γ frequency band (30–80 Hz) and are associated with higher brain functions such as learning and memory (Howard et al., 2003). γ oscillations can be recorded in many brain regions including the hippocampus, olfactory bulb, thalamus and neocortex (Mainy et al., 2007). Generated by the coordinated interaction of excitatory and inhibitory neurons within neural networks (van Vugt et al., 2010), γ oscillations are thought to provide a time frame for synchronized firing of principal neurons (Fisahn et al., 1998; Liu et al., 2004; Fries, 2007), which is critical for the integration of synaptic signals and facilitates synaptic plasticity (Wespatat et al., 2004; Bikbaev and Manahan-Vaughan, 2008).

Ethanol, the most commonly abused drug in humans easily passes the blood-brain barrier and interferes with brain function and behavior. Acute ethanol intoxication may present with symptoms of impaired mental and physical abilities at the blood concentrations >0.25% (~54 mM). Ethanol inhibits the NMDA-type glutamate receptor (NMDAR) (Peoples et al., 1997; Ferrani-Kile et al., 2003) and enhances GABAergic synaptic inhibition (Ariwodola and Weiner, 2004), which causes an imbalance of excitation and inhibition within the neuronal network, which may affect network properties such as γ oscillations. Indeed, ethanol at a moderate dose (0.8 g/kg, ~16 mM) alters stimulus-evoked γ oscillations in the human visual cortex and motor cortex (Campbell et al., 2014). Furthermore, ethanol withdrawal induces a rebound facilitation of γ oscillations in the rat cortex (Cheaha et al., 2014). However, it is not clear whether ethanol modulates γ oscillations at the local network level and, if so, by which the cellular mechanisms.

Acute ethanol exposure affects neuronal membrane receptors and intracellular kinases. At intoxicating concentrations (50–100 mM), acute ethanol activates type 1 dopamine receptor (DR1), adenylate cyclase and cAMP dependent protein kinase A (PKA) (Rabin et al., 1992; Lovinger, 2002; Ferrani-Kile et al., 2003; Moonat et al., 2010; Coller and Hutchinson, 2012), a signaling pathway closely related to synaptic plasticity (Eftekharzadeh et al., 2012; Nassireslami et al., 2013). Acute ethanol can also activate other intracellular kinases such as Akt/protein kinase B (PKB) signaling pathway (Carter et al., 2008; Neasta et al., 2011; Zeng et al., 2012) and glycogen synthase kinase 3 beta (GSK3β), both are multifunctional serine/threonine kinases (French and Heberlein, 2009; Luo, 2009; Zeng et al., 2012; Shah et al., 2015), required for synaptic plasticity (Horwood et al., 2006; Ochs et al., 2015). The involvement of these kinases in ethanol neurotoxicity provides a potential target for therapeutic efforts. Ethanol neurotoxicity can be reduced for example via inhibition of GSK3β activity (Shah et al., 2015).

In this study, we investigated the effect of acute ethanol exposure on kainate-induced γ oscillations and explored possible mechanisms for γ oscillation modulation related to membrane receptors and intracellular kinases.

## Materials and methods

### Animals

All experimental procedures were approved by the Ethics Committees at Xinxiang Medical University for the Care and Use of Laboratory Animals, and all efforts were made to minimize animal suffering and reduce the number of animals used. Electrophysiological studies were performed on hippocampal slices prepared from Sprague Dawley rats (male, 4–5 week-old). Animals were anesthetized by intraperitoneal injection of Sagital (sodium pentobarbitone, 100 mgkg^−1^, Rhône Mérieux Ltd, Harlow, UK). When all pedal reflexes were abolished, the animals were perfused intracardially with chilled (5°C), oxygenated cutting solution containing (in mM): 225 sucrose, 3 KCl, 1.25 NaH_2_PO_4_, 24 NaHCO_3_, 6 MgSO_4_, 0.5 CaCl_2_ and 10 glucose (305 mosmol l^−1^). Horizontal brain slices (400 μm) containing the ventral hippocampus were cut at 4–5°C in cutting solution, using a Leica VT1000S vibratome (Leica Microsystems UK, Milton Keynes, UK).

### Electrophysiological recording, data acquisition, and analysis

For extracellular field recordings, two hippocampal slices were transferred to an interface recording chamber. The slices were maintained at a temperature of 32°C at the interface between artificial cerebrospinal fluid (ACSF) and warmed humidified carbogen gas (95% O_2_–5% CO_2_). The ACSF contained (in mM): 126 NaCl, 3 KCl, 1.25 NaH_2_PO_4_, 24 NaHCO_3_, 2 MgSO_4_, 2 CaCl_2_ and 10 Glucose. The slices were allowed to equilibrate for 1 h prior to recording. Extracellular field potentials were recorded from stratum pyramidale of area CA3c with glass microelectrodes (resistance 2–5 MΩ) and amplified with an Axoprobe 1A amplifier (Axon Instruments, Union City, CA, USA) and a Neurolog system NL106 AC/DC amplifier (Digitimer Ltd., Welwyn Garden City, UK). Data were band-pass filtered between 0.5 Hz and 2 kHz, using a Neurolog system NL125 filter (Digitimer Ltd., Welwyn Garden City, UK). Electrical interference from the mains supply was eliminated from extracellular recordings online with the use of 50 Hz noise eliminators (HumBug; Digitimer Ltd.). The data were digitized at a sample rate of 5–10 kHz using a CED 1401 plus ADC board (CED, Cambridge, UK).

Data were analyzed off-line using Spike 2 software (CED, Cambridge, UK). Power spectra were generated to provide a quantitative measure of the frequency components in a stretch of recording, where power, as measure of the oscillation strength, was plotted as function of frequency. Power spectra were constructed for 60 s epochs of extracellular field recordings using a fast Fourier transform algorithm (Hanning window). The parameters used for measuring the oscillatory activity in the slice were peak frequency and γ power (computed area under the power spectrum between the frequencies of 20 and 60 Hz).

All statistical tests were performed using SigmaStat software (Systat Software Inc, San Jose, USA). Results are expressed as mean ± standard error of mean. Statistical significance for comparison between two groups or among three groups was determined using tests described in the text or in the figure legends, as appropriate. Measures were considered statistically significant, if *P* < 0.05.

### Drugs

The NMDA receptor antagonist D-(−)-2-amino-5-phosphonopentanoic acid (D-AP5), the DR1/5 antagonist R(+) -7-Chloro-8-hydroxy-3-methyl-1-phenyl-2,3,4,5-tetrahydro-1H-3-benzazepine hydrochloride (SCH23390), the DR2/3/4 antagonist (S)-3,5-Dichloro-N-[(1-ethyl-2-pyrrolidinyl)methyl]-2,6-dih ydroxybenzamide hydrobromide (raclopride), the PKA inhibitor N-[2-(p-Bro mocinnamylamino)ethyl]-5-isoquinolinesulfonamide dihydrochloride (H89), the ERK1/2 inhibitor 1,4-Diamino-2,3-dicyano-1,4-bis(o-aminophenyl mercapto)butadiene monoethanolate (U0126) and the PI3 kinase inhibitor 11-(acetyloxy)-1S,6bR,7,8,9aS,10,11R,11bR-octahydro-1-(methoxymethyl)-9a,11b-dimethyl-3H-furo[4,3,2-de]indeno[4,5-h]-2-ben zopyran-3,6,9-trione (wortmannin) were purchased from Tocris Cookson Ltd (Bristol, UK). The Akt (PKB) inhibitor triciribine (TCBN), the GSK3β inhibitor SB415286 and the kainate receptor agonist kainate were obtained from Sigma-Aldrich (UK). Stock solutions, at thousant times the final concentration, were made up in water, except for NBQX which was dissolved in dimethylsulphoxide, and stored in individual aliquots at −20°C. Final solutions were prepared freshly on the day of the experiment.

## Results

### The effect of acute ethanol exposure on KA -induced γ oscillations

**Figure 1:**
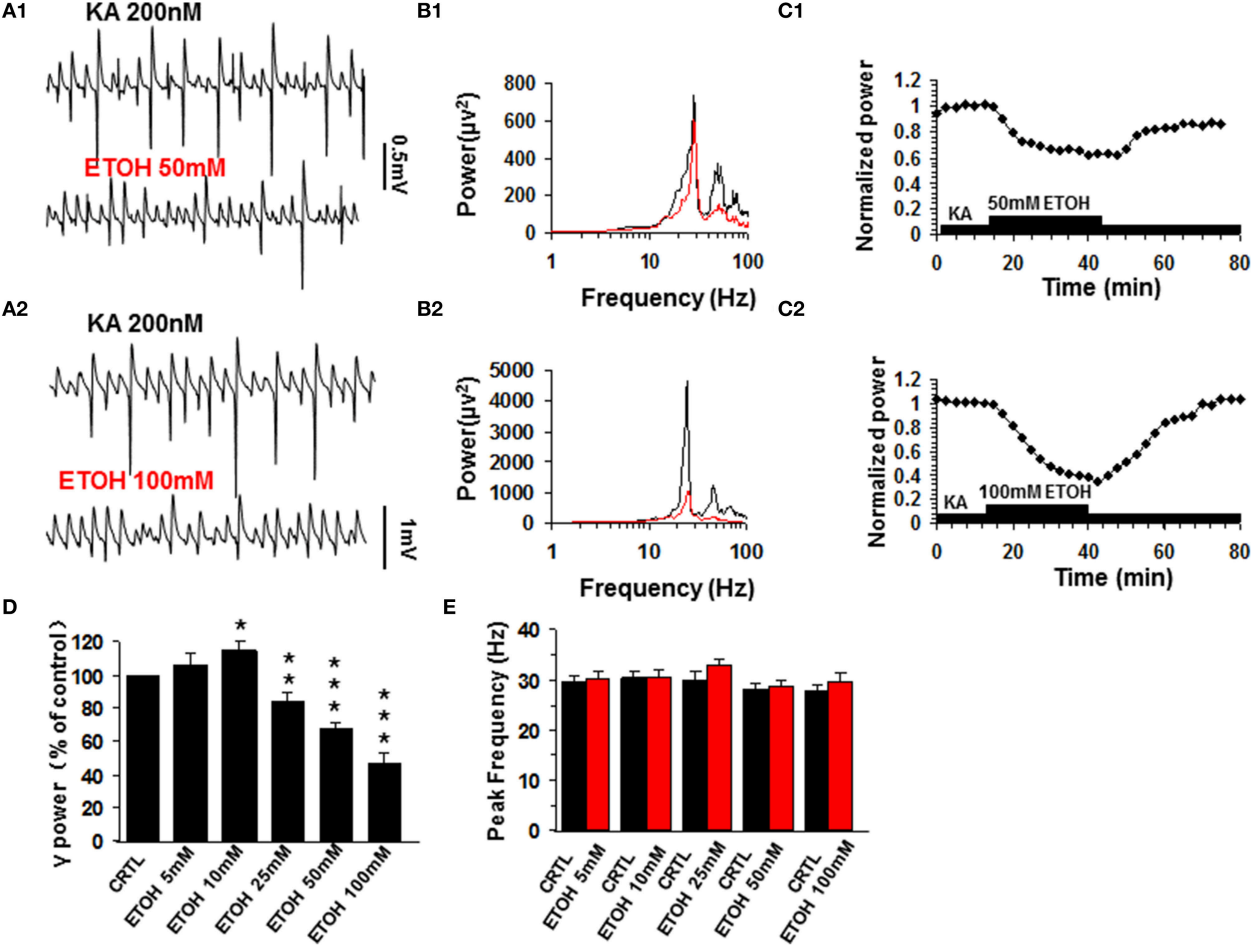
**The effect of ethanol (ETOH) on kainate-induced γ oscillations**. **(A)** Field potential recordings from the CA3 stratum pyramidale, before and after application of 50 mM ethanol **(A1)** or 100 mM ethanol **(A2)**. **(B)** Power spectra corresponding to **(A1,A2)**. **(C)** The time courses of γ power (normalized to the average γ power in the last 5 min before ethanol application (baseline) shows a reversible reduction of γ power. **(D)** γ power as % of baseline γ power for different concentrations of ethanol. (^*^P < 0.05; ^**^P < 0.01; ^***^P < 0.001, compared with baseline, paired *t*-test, *n* = 12, 11, 8, 8, and 10 for 5, 10, 30, 50, and 100 mM ETOH, respectively). **(E)** Peak frequency of γ oscillations in control and for the different concentrations of ethanol (*n* = 8).

Similar to previous reports (Traub et al., 2004), kainate (200 nM) induced persistent γ oscillations in the CA3 area of rat hippocampal brain slices. The γ oscillation normally took 50–120 min to reach a steady state and was then stable for hours. When the steady state was reached, a baseline measure of γ power and peak frequency was taken as the average of 5 min before various concentrations of ethanol (5–100 mM) were added to the ACSF. At 5 mM ethanol had no effect on γ oscillations (106.3 ± 7.0% of base line γ power, *P* > 0.05, *n* = 12). Ethanol caused a small increase in γ power at 10 mM (117.4 ± 6.2% of baseline, *P* < 0.05, *n* = 11), but reduced γ power at concentrations ≥25 mM (Figures 1A–C) in a dose-dependent manner (Figure 1D). Upon washout of ethanol, γ power recovered partially or fully (Figure 1C). On average, ethanol suppressed γ power by 19.4 ± 5.3% (*P* < 0.05, *n* = 8) at 25 mM, by 32.6 ± 4.6% (*P* < 0.01, *n* = 8) at 50 mM and by 52.9 ± 8.5% (*P* < 0.001, *n* = 10) at 100 mM. Ethanol had no effect on peak frequency (Figure 1E).

### The effects of dopamine receptor antagonism on ethanol-induced suppression of γ oscillations

**Figure 2:**
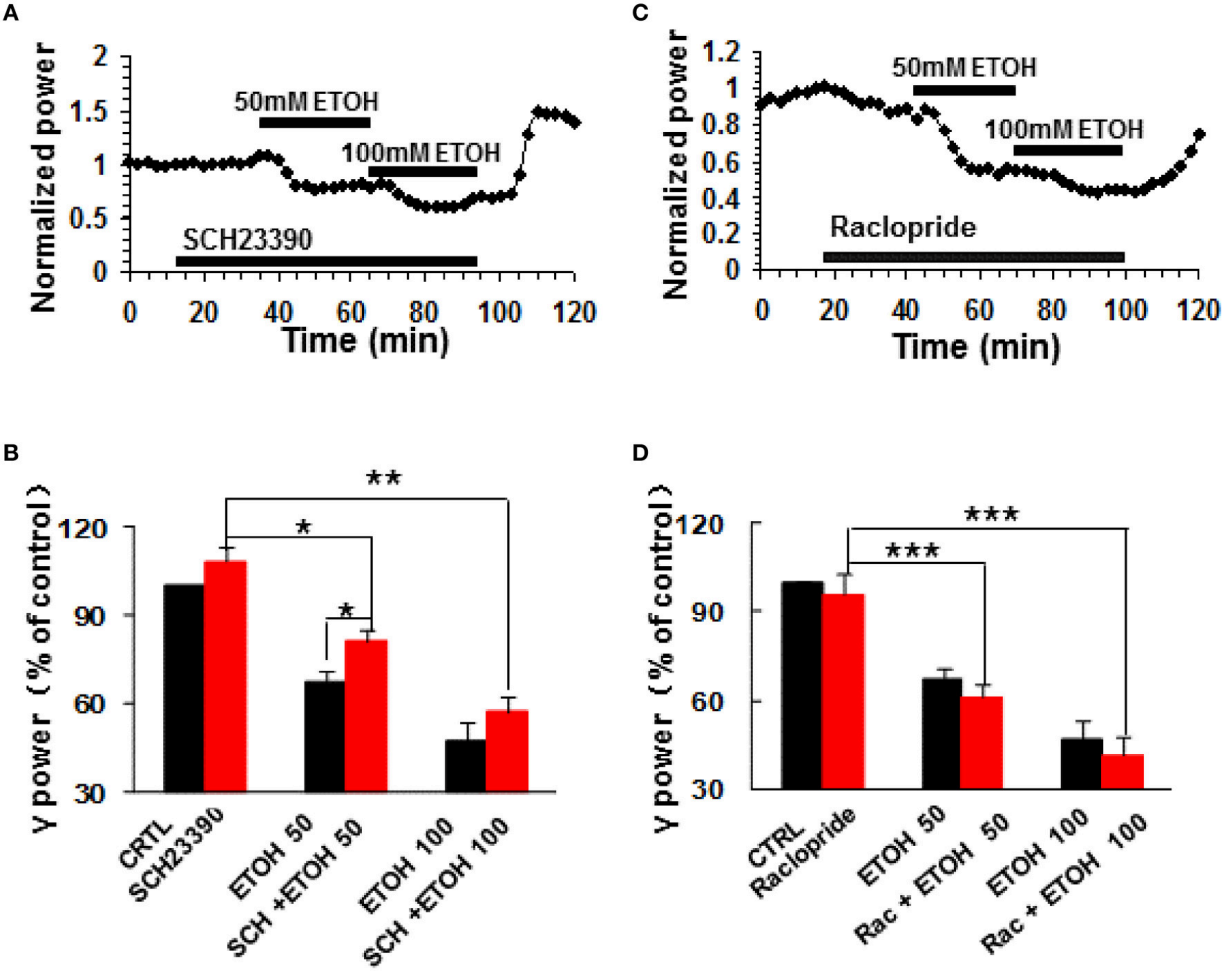
**The effects of dopamine receptor antagonists on ethanol-induced suppression of γ oscillations**. **(A)** Example of the time course of γ power, normalized to the γ power during the 5 min preceding application of the DR1/5 antagonist, SCH23390 (10 μM), with additional application of 50 mM ethanol followed by 100 mM ethanol. **(B)** γ power as % of baseline (γ power only in the presence of kainate as control, CTRL) after application of SCH23390, 50 and 100 mM ethanol (^*^P < 0.05; ^**^P < 0.01; ^***^P < 0.001, compared with SCH23390 baseline, one-way repeated measures Analysis of Variance (RM ANOVA), *n* = 11; ^*^P < 0.05 for comparison between ethanol effects in the presence and absence of SCH23390). **(C)** Example of the effect of ethanol on γ power, in the presence of the DR2/3/4 antagonist raclopride (10 μM) details as in **(A)**. **(D)** γ power as % of baseline for 10 μM raclopride, 50 mM and 100 mM ethanol (*n* = 8). Details as in **(B)**.

To determine whether dopamine receptor (DR) activation contributes to the modulation of γ oscillations by ethanol, we tested the roles of selective DR1/5 antagonists SCH23390 and DR2/3/4 antagonist raclopride. SCH23390 (10 μM) had no effect on γ power (108.4 ± 4.4% of baseline, *P* > 0.05 vs. control, *n* = 11, Figures 2A,B). Subsequent application of ethanol (50 mM) caused a small decrease in γ power (by 18.9 ± 4.1% compared to SCH23390 baseline, *P* < 0.05, *n* = 11, Figure 2A). This decrease was smaller than that induced by 50 mM ethanol alone (Student *t*-test *P* < 0.05). Application of 100 mM ethanol reduced γ power by 42.8 ± 4.9% (*P* < 0.01, *n* = 11). This reduction was not different from that of 100 mM ethanol alone. These results show that DR1/5 antagonist SCH23390 significantly reduced 50 mM but not 100 mM ethanol-induced suppression of γ oscillations.

DR2/3/4 antagonist, raclopride (10 μM) had no effect on control γ power (4.2 ± 6.7% change, *P* > 0.05 vs. baseline, *n* = 11, Figures 2C,D). Raclopride did not change the suppressing effect of 50 mM ethanol (38.8 ± 4.6% decrease, *P* > 0.05 vs. that of 50 mM ethanol alone, *n* = 8, Figure 2D), or 100 mM ethanol (58.3 ± 5.9% decrease, *P* > 0.05 vs. that of 100 mM ethanol, *n* = 8; Figure 2D).

These results indicate that DR1 but not DR2/3/4 are involved in ethanol-induced suppression of γ oscillations.

### The effect of PKA inhibition on ethanol-induced suppression of γ oscillations

**Figure 3:**
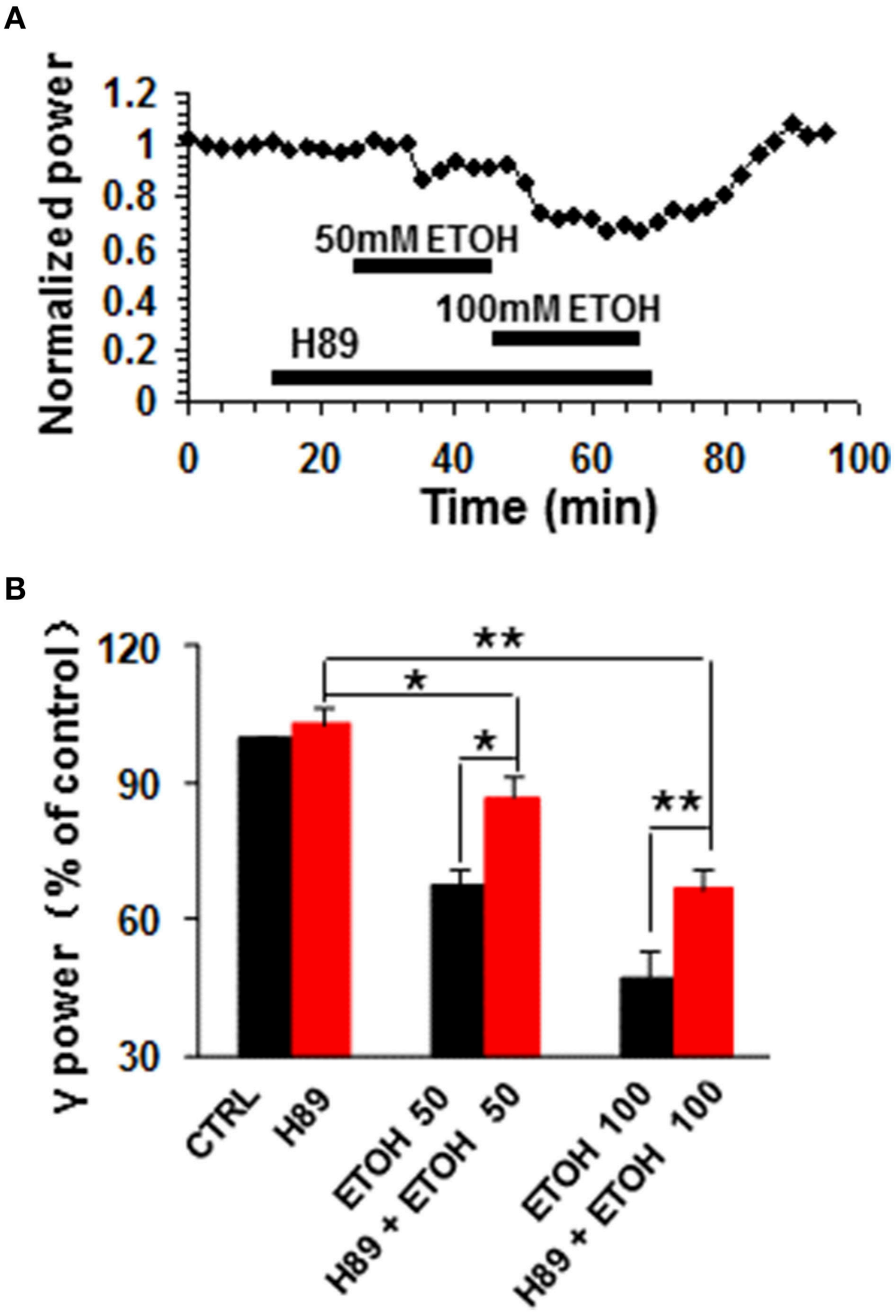
**The effects of PKA inhibitor on ethanol-induced suppression of γ oscillations**. **(A)** Example of the effect of ethanol on γ power in the presence of the PKA inhibitor H89 (10 μM). Details as in Figure 2A. **(B)** γ power as % of baseline for H89, 50 and 100 mM ethanol (*n* = 8). ^*^P < 0.05; ^**^P < 0.01. Details as in Figure 2B.

H89, a potent PKA inhibitor, had no effect on control γ power, but reduced the ethanol-induced suppression of γ oscillations (example in Figure 3A). On average, H89 (10 μM) caused 2.8 ± 3.6% change in γ power (*P* > 0.05 vs. baseline, *n* = 10, Figure 3B). Subsequent ethanol (50 mM) caused a 13.4 ± 4.9% decrease (*P* < 0.05 vs. H89 baseline, *n* = 10) in γ power (Figures 3A,B), which was significantly less than that of 50 mM ethanol alone (Student *t*-test, *P* < 0.05). In the presence of H89 the 100 mM ethanol-induced reduction of γ power was 33.4 ± 4.3% (*P* < 0.01 vs. H89 baseline, *n* = 10), which was smaller than that of 100 mM ethanol alone (Student *t*-test, *P* < 0.05, Figure 3B). These results indicate that PKA is involved in the mechanism underlying ethanol-induced suppression of γ oscillations.

### The effect of PI3K inhibition on ethanol-induced suppression of γ oscillations

**Figure 4:**
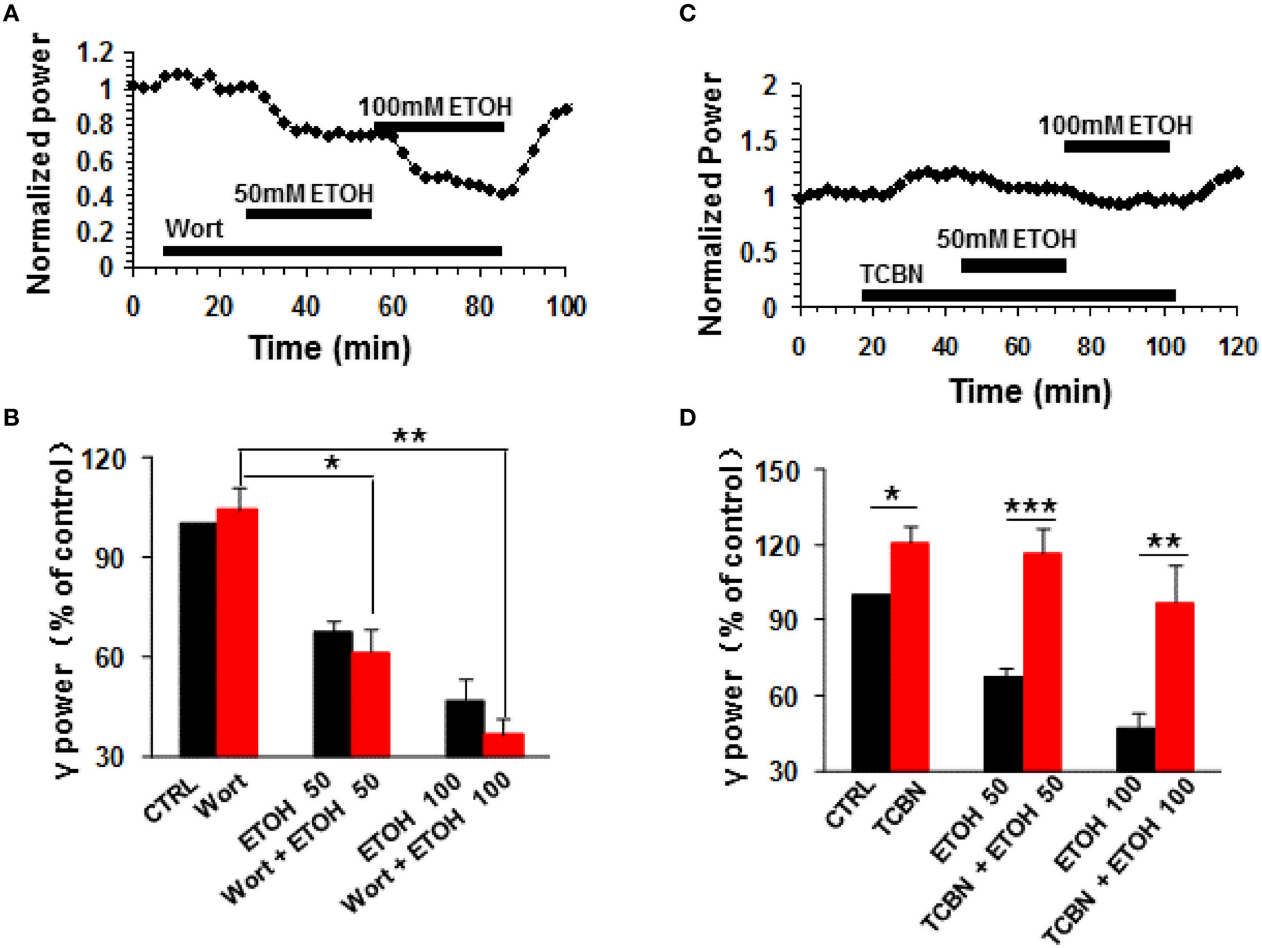
**The effects of PI3-kinase and Akt inhibitors on ethanol-induced suppression of γ oscillations**. **(A)** Example of the effect of ethanol on γ power in the presence of the PI3 kinase inhibitor wortmannin (200 nM). Details as in Figure 2A. **(B)** γ power as % of baseline for wortmannin (Wort), 50 mM ethanol (*n* = 16) and 100 mM ethanol (*n* = 11) Details as in Figure 2B. ^*^P < 0.05; ^**^P < 0.01. **(C)** Example of the effect of ethanol on γ power in the presence of the Akt inhibitor TCBN (5 μM). Details as in Figure 2A. **(D)** γ power as % of baseline for TCBN, 50 mM ethanol (*n* = 9) and 100 mM ethanol (*n* = 9). Details as in Figure 2B. ^*^P < 0.05; ^**^P < 0.01; ^***^P < 0.001.

Phosphatidylinositol-3 kinase (PI3K) converts phosphatidylinositol (3,4)-bisphosphate (PIP2) to phosphatidylinositol (3,4,5)-trisphosphate (PIP3) in the plasma membrane (Yuan and Cantley, 2008), and is involved in many neuronal functions, including neuronal plasticity (Karpova et al., 2006). Wortmannin, a potent and highly selective inhibitor of PI3-kinase had no effect on control γ power (104.3 ± 6.5% of baseline, n.s.) nor ethanol-induced suppression of γ (Figures 4A,B). On average, in the presence of wortmannin (200 nM), 50 mM ethanol caused a 38.7 ± 6.9% decrease (*P* < 0.05, vs. wortmannin baseline, *n* = 8), which was not different from that of 50 mM ethanol alone (Student *t*-test, *P* > 0.05, Figures 4A,B). The reduction induced by 100 mM ethanol in the presence of wortmannin was 63.5 ± 4.7% (*P* < 0.01 vs. wortmannin baseline, *n* = 8, Figure 4B), which was not different from that of 100 mM ethanol alone (Student *t*-test, *P* > 0.05, Figure 4B). These results suggest that PI3 kinase activation was not involved in the ethanol-induced suppression of γ oscillations.

### The effect of Akt inhibition on ethanol-induced suppression of γ oscillations

The serine/threonine kinase Akt is a major kinase of growth factor signaling, can be activated by PI3-kinase or by a PI3-kinase-independent mechanism such as cyclic AMP-dependent protein kinase A (Filippa et al., 1999) or dopamine (Brami-Cherrier et al., 2002). Acute ethanol exposure increases the phosphorylation of Akt (Carter et al., 2008; Zeng et al., 2012). We thus tested whether TCBN, a selective Akt inhibitor, affects the effect of ethanol on γ oscillations. TCBN (5 μM) caused a small increase in γ power (21.1 ± 6.7%, *P* < 0.05, vs. baseline, *n* = 16), but occluded the suppression of γ oscillations by ethanol (example in Figure 4C). The effect of ethanol (50 mM) in the presence of TCBN (16.1 ± 10.2%, *P* > 0.05 vs. TCBN baseline, *n* = 16) was smaller than that in the absence of TCBN (Student *t*-test, *P* < 0.001 vs. 50 mM ethanol alone, Figure 4D). Similarly, the effect of 100 mM ethanol in the presence of TCBN (3.1 ± 14.8%, *P* > 0.05 vs. TCBN baseline, *n* = 11) was smaller than that in the absence of TCBN (Student *t*-test, *P* < 0.01 vs. 100 mM ethanol alone, Figure 4D). Thus, these results suggest that Akt plays a critical role in the ethanol-induced suppression of γ oscillations.

### The effect of GSK3β inhibition on ethanol-induced suppression of γ oscillations

**Figure 5:**
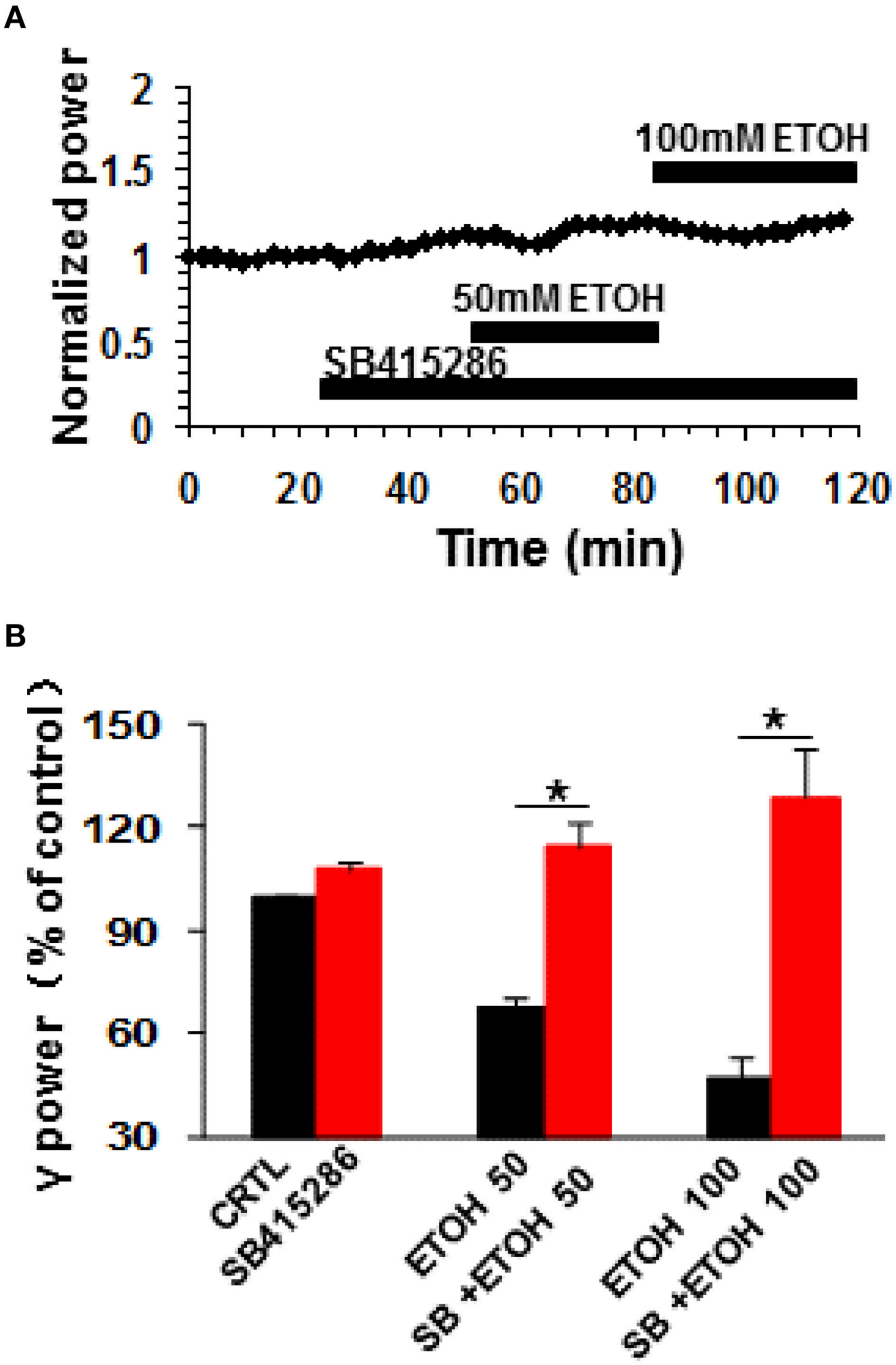
**The effects of GSK3β inhibitor on ethanol-induced suppression of γ oscillations**. **(A)** Example of the effect of ethanol on γ power in the presence of the GSK3β inhibitor SB415286 (5 μM). Details as in Figure 2A. **(B)** γ power as % of baseline for SB415286, 50 and 100 mM ethanol (*n* = 9). Details as in Figure 2B. ^*^P < 0.05.

Glycogen synthase kinase 3beta (GSK3β), a downstream molecule of Akt or PKA, is involved in many physiological and pathological processes, such as synaptic plasticity (Ochs et al., 2015), tau pathology of Alzheimer's disease (Hernandez et al., 2013) and ethanol-induced neuronal excitotoxicity (French and Heberlein, 2009; Luo, 2009; Zeng et al., 2012; Shah et al., 2015). The role of GSK3β in the ethanol-induced suppression was tested with SB415286, a GSK3β inhibitor. SB415286 (5 μM) had no effect on control γ power (7.8 ± 2.5%, *P* > 0.05, vs. baseline, *n* = 9), but completely blocked ethanol-induced suppression of γ (example in Figure 5A). The effect of 50 mM ethanol in the presence of SB415286 (14.3 ± 7.1%, *P* > 0.05 vs. SB415286 baseline, *n* = 9) was completely different from that of ethanol alone (Student *t*-test *P* < 0.001 vs. 50 mM ethanol alone, Figure 5B). Similarly, the effect of 100 mM ethanol (28.9 ± 14.1%, *P* > 0.05 vs. SB415286 baseline, *n* = 9) was different from that in the absence of SB415286 (Student *t*-test, *P* < 0.001 vs. 100 mM ethanol alone, Figure 5B). These results suggest that GSK3β activation is essential for the ethanol-induced suppression of γ oscillations.

### The role of NMDA receptor antagonism on ethanol-induced suppression of γ oscillations

**Figure 6:**
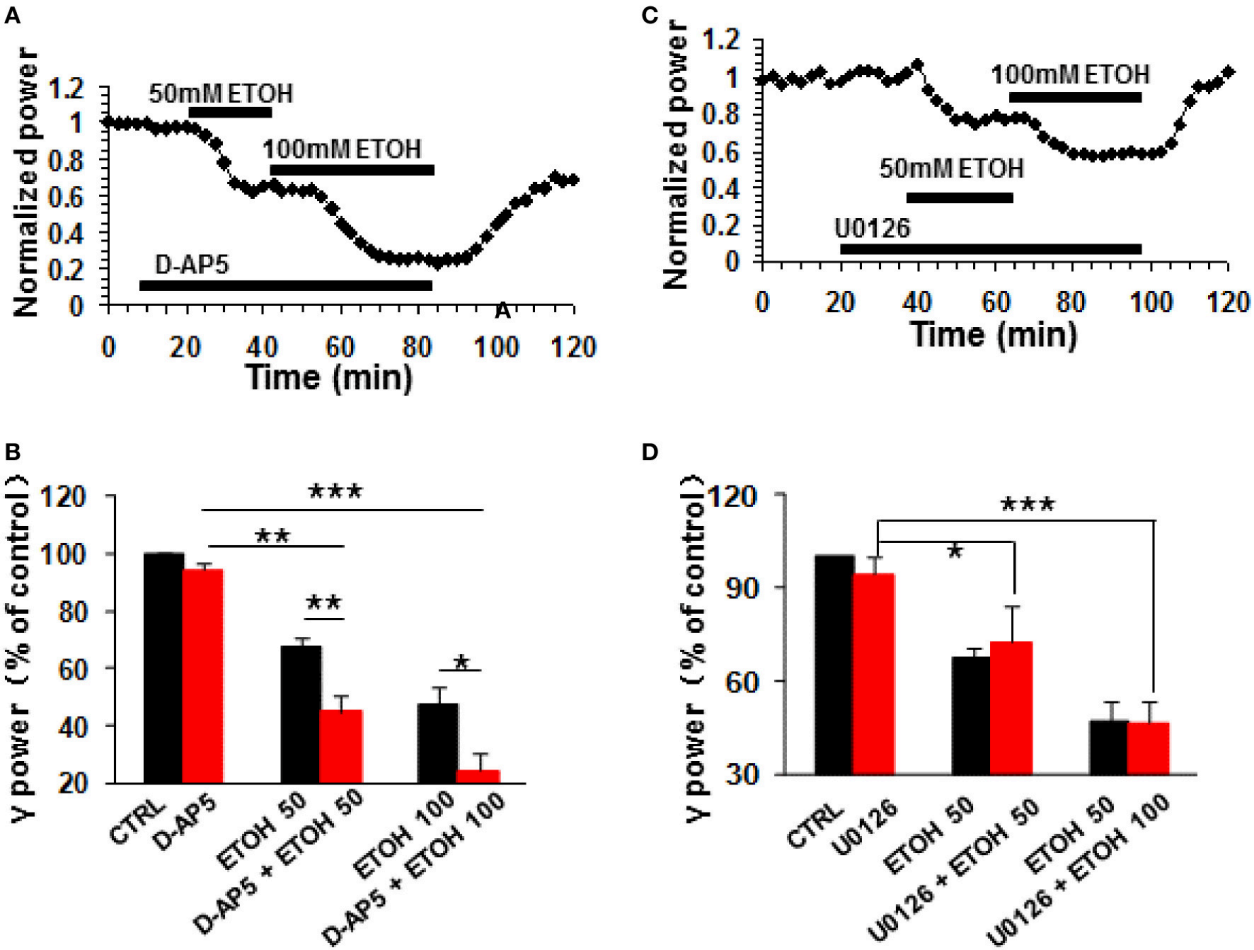
**The NMDA receptor antagonist and ERK inhibitor on ethanol-induced suppression of γ oscillations**. **(A)** Example of the effect of ethanol on γ power in the presence of D-AP5 (10 μM). Details as in Figure 2A. **(B)** γ power as % of baseline for NMDAR antagonist D-AP5, 50 and 100 mM ethanol (*n* = 8). Details as in Figure 2B. ^*^P < 0.05; ^**^P < 0.01; ^***^P < 0.001. **(C)** Example of the effect of ethanol on γ power in the presence of ERK inhibitor U0126 (2.5 μM). Details as in Figure 2A. **(D)** γ power as % of baseline for U0126, 50 and 100 mM ethanol (*n* = 8). Details as in Figure 2B. ^*^P < 0.05; ^***^P < 0.001.

The NMDA receptor antagonist D-AP5 (10 μM) had no effect on control γ power but enhanced the ethanol-induced suppression of γ (example in Figure 6A). On average, 50 mM ethanol caused a 55.1 ± 5.4% decrease in γ power in the presence of D-AP5 (*P* < 0.01 vs. D-AP5 baseline, *n* = 8), which was bigger than the effect of ethanol alone (Student *t*-test, *P* < 0.01 vs. 50 mM ethanol alone, Figure 6B). Similarly, the suppression caused by 100 mM ethanol in the presence of D-AP5 (75.7 ± 6.1% decrease, *P* < 0.001 vs. D-AP5 baseline, *n* = 8) was bigger than that in the absence of D-AP5 (Student *t*-test, *P* < 0.05 vs. 100 mM ethanol alone, Figure 6B). These results suggest that NMDA receptor blockade enhanced ethanol-induced suppression of γ oscillations.

### The effect of MAPK inhibition on ethanol-induced suppression of γ oscillations

The extracellular signal-regulated kinase (ERK1/2) is one of the downstream kinases of NMDA receptors in the hippocampus, controlling the neuroplasticity underlying memory processes and neuronal development (Krapivinsky et al., 2003). U0126, a selective inhibitor of MAPK kinase (MEK1/2), the immediate upstream activator of ERK1/2, inhibits ERK1/2 activation in acute slices (Giovannini et al., 2003). U0126 (2.5 μM) did not affect control γ power (*P* > 0.05, vs. baseline, *n* = 8) or the suppression of γ oscillations by ethanol (example in Figure 6C). In the presence of U0126, 50 mM ethanol caused a 27.5 ± 12.0% (*P* < 0.01, vs. U0126 baseline, *n* = 8) and 100 mM ethanol a 53.6 ± 7.4% (*P* < 0.001, vs. U0126 baseline, *n* = 8) decrease in γ power, neither of which was different from that in the absence of U0126. (Figure 6D). These results suggest that ERK activation is not required for the ethanol-induced suppression of γ oscillations.

## Discussion

In this study, we found that (1) ethanol suppressed hippocampal γ oscillations only at intoxicating concentrations; (2) This effect was reduced by a DR1 antagonist or a PKA inhibitor, completely prevented by Akt and GSK3β inhibitors, but enhanced by NMDA receptor antagonist; (3) MAPK and PI3-kinase inhibitors did not affect the effect of ethanol. These results suggest that the ethanol-induced suppression of γ is dependent on in the activation of DR1, PKA, Akt, and GSK3β and facilitated by the inhibition of NMDA receptors.

### Role of acute ethanol on γ oscillation

Our study shows that ethanol (10 mM) increased γ power without affecting the peak frequency, suggesting that at a low concentration, ethanol may increase network activity. Because IPSCs are crucial for γ oscillations (Cunningham et al., 2003), the enhancement of γ oscillations at low ethanol concentrations can be ascribed to increased phasic IPSC amplitudes (Wan et al., 1996). This is somewhat similar to a recent report that showed ethanol (15 mM) increased peak γ amplitude in human primary visual and motor cortex (Campbell et al., 2014). However, such an increase in γ power is limited in amplitude and is only observed in a narrow range of ethanol concentrations in this study.

**Figure 7:**
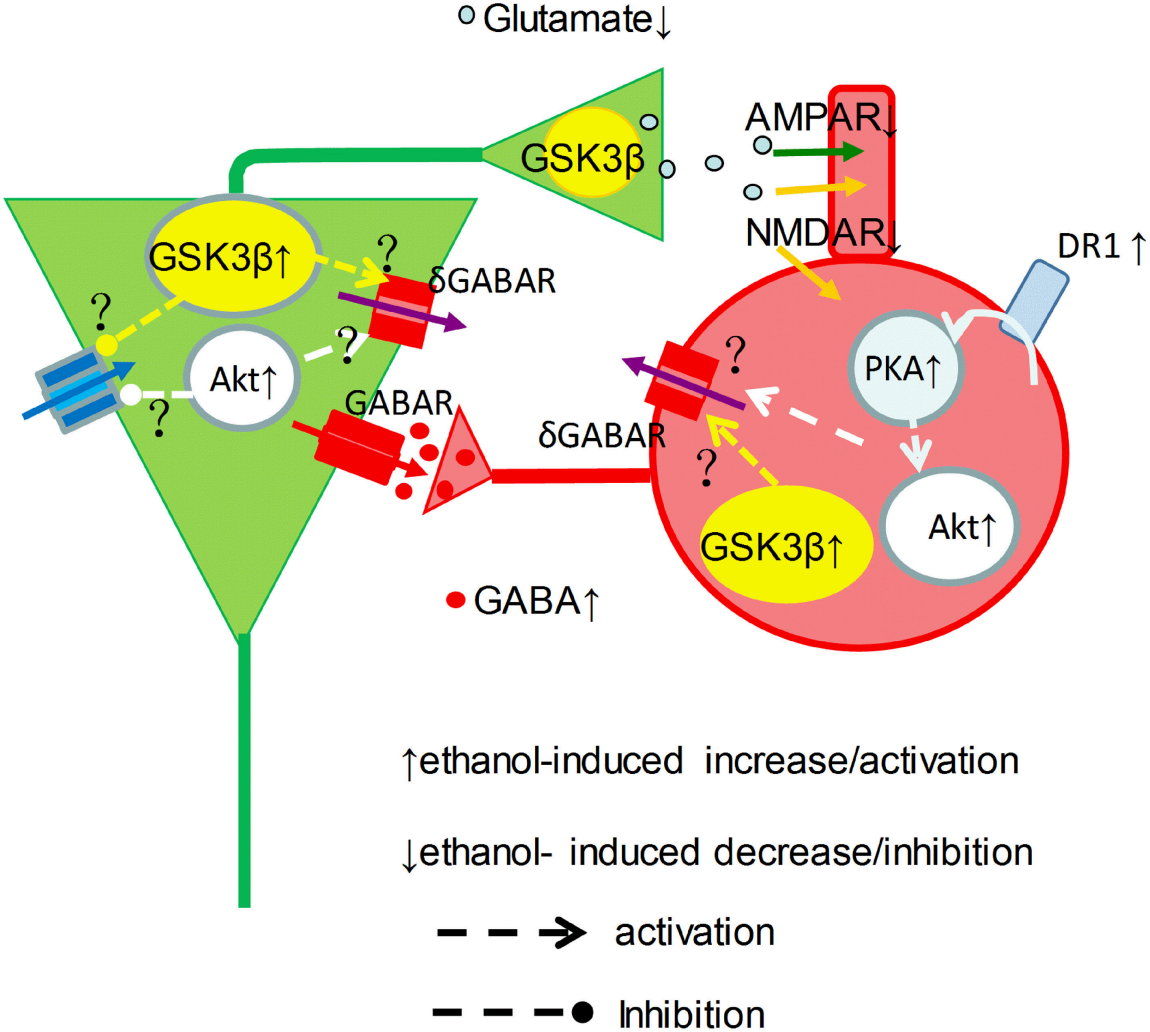
**Graphical representation of proposed ethanol-induced changes to the γ-generating network**. Cellular excitability resulting from a tonic drive by intrinsic properties (blue arrow), NMDAR (orange arrow) and tonic δGABAR (purple arrow), is shaped by phasic GABAR (red arrows) and AMPAR (green arrows) activity in pyramidal neurons (green) and interneurons (red) in a simplified γ-generating network. At intoxicated concentrations, ethanol activates DR1-cAMP-PKA signaling, and causes Akt and GSK3β activation increase, which may increase phasic GABA release. On the other hand, ethanol inhibit NMDAR activity and increase δ GABAR activity, which can be mediated through activation of Akt and GSK3β activation, reducing the excitatory drive and phasic glutamate release, suppressing γ oscillations.

Our data showed a clear picture of reduction of γ power when the concentration of ethanol was ≥25 mM. At intoxicating levels ethanol impairs working memory and perception amongst others (Schweizer et al., 2006), which can be explained by the strong suppression of γ oscillations reported here. Because ethanol even at intoxicated concentration also increases GABA_*A*_ receptor mediated IPSCs (Wan et al., 1996; Wu et al., 2005; Zheng et al., 2016), it remains the question how an ethanol-induced increase in IPSCs would suppress γ oscillations. Ethanol could inhibit the activation or reduce excitatory drive of interneurons by pyramidal cells, since acute ethanol dose-dependently inhibits synaptic transmission (Hendricson et al., 2004; Badanich et al., 2013). Alternatively, ethanol could reduce interneuron excitability due to an increase of tonic inhibition (Wei et al., 2004), resulting from synaptic spillover acting on δ subunit-containing GABAA receptors (Mann and Mody, 2010). It seems that ethanol may enhance GABAergic neurotransmission via increment of interneuron excitability and GABA release, and may decrease glutamatergic neurotransmission via reduction of glutamate release(Hendricson et al., 2004) and inhibition of NMDA receptor function(Lovinger et al., 1990), which causes striking imbalance between excitation and inhibition within neuronal network and impairment of γ oscillations (Figure 7).

### The DR1-PKA pathway is involved in the ethanol-induced suppression of γ oscillations

The reduction of the ethanol-induced suppression of γ oscillations by the PKA inhibitor H89 indicates the involvement of the PKA pathway in the ethanol effect at intoxicating concentrations, which is consistent with the report of acute ethanol induction of PKA activation (Naseer et al., 2010). The sensitivity of the ethanol effect to a DR1 antagonist suggest that the cAMP that drives PKA is in part due to the activation of DR1 (Rex et al., 2008), which are in area CA3 only expressed on interneurons (Gangarossa et al., 2012). Since ethanol causes an increase in dopamine release in the brain, this effect could even be stronger *in vivo* (Deng et al., 2009; Morikawa and Morrisett, 2010; Jerlhag et al., 2011; Theile et al., 2011).

### The role of NMDARs, ERK, and PI3-kinase on the ethanol-induced suppression of γ oscillations

NMDARs are involved in acute ethanol intoxication and addiction-related behaviors (Krystal et al., 2003; Möykkynen and Korpi, 2012). At intoxicating concentrations ethanol causes an inhibition of NMDARs (Lovinger et al., 1990; Proctor et al., 2006; Xu and Woodward, 2006). The role of NMDARs in γ oscillations is debated. NMDARs are not necessary for the phasic synaptic excitation in γ-generating networks (Fisahn et al., 2004), which is in line with our observation that D-AP5 itself had no effect on control γ oscillation. However, NMDAR blockade enhances the ethanol-induced suppression of γ, which may be associated with the contribution of NMDAR to the tonic drive of interneurons (Middleton et al., 2008; Mann and Mody, 2010; Xue et al., 2011). The suppression of γ oscillations may reveal a critical dependence on the NMDAR-mediated drive of interneurons in the presence of ethanol. In line with our observations, a recent study showed that blocking the glycine(B) site of NMDRs potentiated ethanol intoxication (Debrouse et al., 2013).

NMDAR activation mediates the phosphorylation of downstream molecules such as ERK1/2 and PI3-kinase is a central mediator of NMDAR signaling to Erk1/2 (Perkinton et al., 2002; Crossthwaite et al., 2004). However, the lack of effect of inhibitors of MAPK and PI3-kinase on the ethanol-induced suppression of γ oscillations, suggests these kinases are not involved in ethanol-induced suppression of γ, which may be explained by an inhibition of ionotropic NMDAR function.

### The roles of the Akt and GSK3β in the ethanol-induced suppression of γ oscillations

NMDAR stimulation can activate Akt via PI3-kinase signaling (Perkinton et al., 2002; Crossthwaite et al., 2004). Interestingly, Akt can be also activated via dopamine receptor mediated cAMP-PKA signaling, a PI3-kinase independent mechanism (Filippa et al., 1999; Brami-Cherrier et al., 2002). Since blocking PI3-kinase had no effect on ethanol-induced suppression of γ oscillations, Akt activation is likely through DR-PKA signaling (Filippa et al., 1999; Brami-Cherrier et al., 2002). The initial increase in γ power and the complete occlusion of the ethanol-induced suppression of γ oscillations by an Akt inhibitor suggests that Akt activation, due to acute ethanol exposure (Neasta et al., 2011; Zeng et al., 2012) inhibits γ-generating network through yet unknown pathways (Figure 7).

GSK3β is involved in ethanol-induced neurotoxicity (French and Heberlein, 2009; Luo, 2009; Zeng et al., 2012; Shah et al., 2015). The complete occlusion of the ethanol-induced suppression of γ oscillations by a GSK3β inhibitor indicates that GSK3β activity is crucially involved in the γ oscillation suppression associated with intoxicating effect of ethanol. This is supported by the observation that ethanol increases GSK3β activity via dephosphorylation of GSK3β at serine 9 (Liu et al., 2009). At this moment it is not clear how GSK3β activity affects intrinsic and or tonic synaptic activity in the γ-generating network. Interestingly, GSK3β negatively modulates presynaptic glutamate release (Zhu et al., 2010), which could contribute to the ethanol-induced suppression of γ oscillations (Figure 7).

Although GSK3β can be phosphorylated at Ser9 by multiple kinases such as Akt, ERK and PKA, such a phosphorylation of GSK3β will only inactivate GSK3β (Li et al., 2000; Jo et al., 2011; Yamaguchi et al., 2012), and thus cannot explain an ethanol-induced activation of GSK3β. Ethanol-induced GSK3β activation may be explained by the decreased levels of inhibitory GSK3β phosphorylation at the Ser9 residue (Liu et al., 2009). Alternatively, GSK3β is also capable of being regulated by an activation pathway. Activation of GSK3β is accomplished by phosphorylation at tyrosine 216 (Tyr216), but whether ethanol can induce GSK3β phosphorylation at Tyr216 remains to be further determined.

### The possible synaptic sites of ethanol-induced suppression of γ oscillations

Acute ethanol increases the probability of GABA release in hippocampal CA1 pyramidal neurons and in the central nucleus of the amygdala (Wu et al., 2005; Li et al., 2014; Valenzuela and Jotty, 2015). Ethanol depresses the frequency, but not the amplitude of miniature excitatory post-synaptic currents (mEPSCs) in CA1 neurons, suggesting that ethanol reduces pyramidal cell excitability and thus phasic activation of interneurons (Badanich et al., 2013). However, previous study also reported that ethanol (25–75 mM) dose-dependently inhibits mEPSC amplitude and frequency, suggesting that the acute effects of ethanol on NMDAR signaling at hippocampal synapses are multifocal in nature (Hendricson et al., 2004).

The intracellular kinases such as PKA and GSK3β are located at both pre- and post-synaptic sites, which regulate neuronl function including excitability and neurotransmitter release (Zhu et al., 2010; Park et al., 2014). GSK3β-mediated inhibition of presynaptic glutamate release may contribute to ethanol-induced suppression of γ oscillations, due to ethanol-induced activation of GSK3β (Zhu et al., 2010).

### Clinical significance of the ethanol-induced suppression of γ oscillations

The suppression of γ oscillations by ethanol is likely to contribute to the behavioral effects associated with ethanol intoxication. Understanding the mechanisms underlying ethanol intoxication may provide cues for reversing it. Our observations imply that enhancing NMDAR could be a strategy to reduce acute ethanol intoxication. However, whereas glycine(B) site blockade potentiated ethanol intoxication, exogenous glycine(B) site activation failed to produce the hypothesized reduction in ethanol intoxication, perhaps because of saturating endogenous glycine levels (Debrouse et al., 2013). Other avenues to pursue could be the inhibition of the Akt/GSK3β signaling. Indeed inhibition of the Akt/GSK3β signaling by serotonin deficiency causes a reduced sensitivity to the intoxicating effect of ethanol (Sachs et al., 2014).

Our observations may also provide cues for therapies preventing ethanol withdrawal symptoms. Ethanol withdrawal can cause delirium tremens, characterized by hallucinations or even seizures. Withdrawal of ethanol after chronic use causes a strong increase in γ oscillations in rats (Cheaha et al., 2014). Enhanced γ oscillations are associated with psychosis (Hirano et al., 2015) and can even trigger seizures (de Curtis and Avoli, 2016). Withdrawal-induced increase in γ oscillations could be explained by a rebound effect caused by an adaptation to a chronic suppression of γ-generating networks. In theory, a temporary suppression of γ oscillations during ethanol withdrawal, e.g., by facilitation of the Akt/GSK3β signaling or NMDAR antagonists may alleviate withdrawal symptoms. Interestingly, increasing serotonin availability with fluoxetine reduced the withdrawal-induced γ oscillation rebound (Cheaha et al., 2014) and withdrawal symptoms in rats (Uzbay et al., 2004). Furthermore, the NMDAR antagonist ketamine was shown to be beneficial in the treatment of ethanol withdrawal symptoms (Wong et al., 2015).

Further understanding of the pathways involved in the effect of intoxicating levels of ethanol on γ oscillations is needed to develop more effective therapies for reversing acute toxicity as well as withdrawal symptoms.

## Author contributions

JW performed the experiments, analyzed the data and wrote the paper; JZ, ZL, FG, and YW performed the experiments; XW, RZ, and MV analyzed the data; CL designed the experiments, performed the experiments and wrote the paper.

### Conflict of interest statement

The authors declare that the research was conducted in the absence of any commercial or financial relationships that could be construed as a potential conflict of interest.
